# The Growing Incidence of Bullous Pemphigoid: Overview and Potential Explanations

**DOI:** 10.3389/fmed.2018.00220

**Published:** 2018-08-20

**Authors:** Khalaf Kridin, Ralf J. Ludwig

**Affiliations:** ^1^Department of Dermatology, Rambam Health Care Campus, Haifa, Israel; ^2^Lübeck Institute of Experimental Dermatology, University of Lübeck, Lübeck, Germany

**Keywords:** bullous pemphigoid, epidemiology, explanation, review of literature, incidence, DPP-4 inhibitors, longevity

## Abstract

Bullous pemphigoid (BP) is the most common type of subepidermal autoimmune bullous diseases. BP characteristically affects the elderly and is seen mainly in patients older than 70 years. While the annual incidence of BP has been estimated to be between 2.4 and 23 cases per million in the general population, it rises exponentially to 190–312 cases per million in individuals older than 80 years. In addition, a growing body of evidence reports a remarkable trend of increased incidence of BP, showing a 1.9- to 4.3-fold rise over the past two decades. This demonstrable increase warrants a higher awareness of the increased risk to develop BP. This review summarizes the current understanding of the epidemiological features of BP and sheds light on the putative explanations for its growing incidence.

## The general epidemiology of BP

Bullous pemphigoid (BP) is the most common type of subepidermal autoimmune bullous diseases (1). The disease develops characteristically in the elderly, particularly in patients older than 70 years (2–5). The annual incidence of BP has been estimated to range between 2.4 and 21.7 new cases per million population in different populations worldwide (2, 3, 6–11). An even higher annual incidence of 42.8 cases per million population was reported in the United Kingdom (UK), although this report may be interpreted with caution as it is based on a computerized longitudinal general practice database (12). Also based on health insurance data, the prevalence of BP has recently been estimated at 259 per million population in Germany, i.e., ~21,000 patients with BP lived in Germany as for 2014 (13). Despite this increase, BP is still considered as an orphan disease, i.e., < 5 in 100,000 persons are affected by the disease (14).

BP is traditionally considered as a disease of the elderly population. The mean age of presentation ranges between 66 and 83 years in different cohorts across the globe (9, 15). The incidence rises exponentially with age, culminating at 190 to 312 cases per million per year in individuals older than 80 years of age (3, 11, 12, 16). Conversely, BP is rarely encountered in individuals younger than 50 years, with the reported incidence rates usually lower than 0.5 cases per million population in this age category (3, 8, 12, 17). This is atypical for an autoimmune disease, caused by an autoantibody response toward the hemidesmosomal proteins BP180 and BP230 (18–22), as autoimmune diseases usually arise during young adulthood (23–25).

An evident female preponderance was noted in the majority of studies, with a female-to-male ratio ranging between 1.04 and 5.1 (2, 4, 6, 7, 9, 12, 15, 26–28). Several studies found that the incidence rate seems to be higher in women until the age of 75, but thereafter the incidence is higher in men (3, 9, 17).

## Overview of the increasing trend in different regions in the last two decades

A growing incidence ranging from 1.9- to 4.3-fold in the past two decades has been reported in recent data from the UK, France, Germany, and Israel (Table 1) (2, 9, 12, 17, 28).

**Table 1:** Increase in bullous pemphigoid incidence across different populations.

**Country**	**First incidence rate**	**Second incidence rate**	**Third incidence rate**	Increase*	**References**
France	6.7 (1986–1992)	21.7 (2000–2005)		3.3-fold within 14 years	(2, 9)
Germany	6.1 (1989–1997)	6.6 (1989–1994)	13.4 (2001–2002)	2.2-fold within 8 years	(8, 28, 29)
United Kingdom	10.0 (1985)	14.0 (1991–2001)	42.8 (2001–2004)	4.3 within 17 years	(11, 12, 30)
Israel	7.6 (2000–2005)	12.6 (2006–2010)	14.3 (2011–2015)	1.9 increase within 10 years	(17)

Incidence rates refer to cases per million population per year. Figures in brackets indicate the years when the incidence was determined. *The period in which the increase occurred was estimated by calculating the difference between the midpoint of the first and last follow-up periods.

In a retrospective cohort study, the annual incidence of BP was estimated at 21.7 cases per million population throughout the years 2000–2005 in three French regions: Haute-Normandie, Limousin, and Champagne-Ardennes (9). This figure represents more than 3-fold increase relative to the annual incidence previously estimated in three adjacent French regions between the years 1986–1992: Limousin, Touraine, and Picardie, with very similar demographic characteristics (6.7 cases per million population) (2). This rise corresponds to an average increase of approximately one case per million per calendar year (9).

In accordance to the French study, very similar incidence rates had been concurrently calculated in two German regions during a similar period, namely Lower Franconia (6.1 cases per million population) and Northwestern Bavaria (6.6 cases per million population), between the years 1989–1997 and 1989–1994, respectively (28, 29). Bertram et al. (8) reported a 2.2-fold increase in annual incidence rate in Lower Franconia in 2001–2002 (13.4 cases per million population) as compared to the previous study.

A consistent rise of the annual incidence rates of BP has been reported in the UK during the last two decades; from a nadir of 10.0 cases per million population in 1985 (30), to 14.0 cases per million population during 1991–2001 (11), and up to 42.8 cases per million population during the 2001–2004 (12). However, the last study may be biased by overestimation as was previously discussed (12).

A recent population-based study investigating the epidemiology of BP in Northern Israel revealed a 1.9-fold increasing annual incidence: from 7.6 to 14.3 cases per million population in the calendar periods 2000–2005 and 2011–2015, respectively. This rise applied to both of the major ethnic populations residing in the region: Jews and Arabs (17).

## Putative explanations for this surge

### Increasing life expectancy of the populations

Global mean life expectancy increased substantially by 5.5 years between 2000 and 2016, the most rapid increase observed since the 1960s (31). Over the past 15 years, a 2.9 year increase in life expectancy has occurred in the European Union countries, rising from 77.7 to 80.6 years; the rise was 2.4 years for women and 3.4 years for men (32). This rapid rise was attributed to several putative factors including the improved lifestyles, rising living standards, better education, and advanced healthcare and medicine (32). Given the increasing incidence rate of BP with age, the higher longevity account for part of the increased overall incidence of BP throughout the years.

In countries where rising incidence of BP was reported, a parallel rise in life expectancy was observed. In France and the UK between 1980 and 2000, life expectancy rose from 74.1 to 79.2 years and from 73.7 to 78.0 years, respectively (32, 33). In 2015, life expectancies in these two countries further increased to 82.4 and 81.0 years, respectively (32). In Germany, the life expectancy increased consistently, rising from 73.1 in 1980 to 78.3 in 2000 and reaching 80.7 in 2015 (32). When examining fluctuations in the life expectancy throughout the relevant period in Israel, a 3 years increase was identified; from 79.0 in 2000 to 82.1 in 2015 (33). Considering the aging trend of the European population, even more individuals are expected to develop BP in the coming decades.

### Increasing incidence of disabling neurological conditions

A large body of evidence gathered within the past decade suggests a high association between neurological disorders and BP. The prevalence of neurological comorbidities among patients with BP ranges between 28 and 56% (34–37). In addition, neurological conditions were found to be an independent risk factor for the subsequent development of BP as revealed by several well-designed observational studies (16, 38–40). The presence of coexisting neurological disease at the onset of BP was found to be a bad prognostic factor (39, 41–43).

The burden of neurological disease has grown remarkably within the past few decades (44). The increasing number of individuals affected by these diseases has mainly been associated with the aging of the population and population growth (44). There was a large rise in the absolute numbers of prevalent cases of Alzheimer's disease and other dementias (44, 45). In addition, an increasing incidence of stroke (46), epilepsy (47), and intracranial malignancies in elderly people (48), as well as growing prevalence of multiple sclerosis (49, 50) have been reported in different regions. The increasing burden of these neurological conditions, found to be an established risk factor for BP, may account for part of the rising incidence of BP.

One of the widely accepted explanations for this association is the cross-reactivity between the neuronal and epithelial isoforms of BP Antigen-1 (BP230), which are both encoded by dystonin gene (*DST*) (51–55). Mice with mutations in this gene developed severe dystonia and sensory nerve degeneration, further supporting this concept (56). Additionally, a large amount of evidence suggests that BP180 may also be expressed in certain components of the central nervous system (54, 55).

### Increasing use of certain culprit drugs

#### Dipeptidyl-peptidase IV inhibitors

Growing evidence suggests that dipeptidyl-peptidase IV inhibitors (DPP4i), new anti-hyperglycemic oral agents used to treat type 2 diabetes mellitus, may be implicated in the development of BP (57, 58). Sitagliptin was the first DPP4i agent to gain US Food and Drug Administration approval in 2006, followed by vildagliptin (2007), linagliptin (2011), and alogliptin (2013) (57). Since the approval of these agents, their administration has become more widespread as either monotherapy or in conjunction with insulin or other oral antihyperglycemic medications.

The role of DPP4i in triggering BP was grounded mainly on case reports (59–66) and national pharmacovigilance database analyses (67, 68) until well-designed controlled observational studies were recently conducted (57, 58). Benzaquen et al. (57) reported that DPP4i intake was associated with an increased risk for triggering BP (adjusted OR, 2.64), with vildagliptin being implicated with the highest risk (adjusted OR, 3.57). Varpuluoma et al. (58) found that DPP4i administration was associated with 2.2-fold increased risk for BP (adjusted OR, 2.19), particularly with vildagliptin (adjusted OR, 10.4). The aforementioned studies were underpowered to analyze the association between BP and linagliptin, which is most likely due to the limited number of cases under treatment with this new agent. Kridin and Bergman observed that overall DPP4i intake was associated with a 3-fold increased risk for BP (adjusted OR, 3.16). The adjusted ORs of vildagliptin and linagliptin were 10.67 and 6.65, respectively. Furthermore, a recent Greek retrospective cohort study observed a 38.4% increase in the prevalence of patients with comorbid type 2 diabetes mellitus in a cohort of 130 patients with BP. This rise was ascribed mainly to the growing prescription of DPP4i (69). Interestingly, DPP4i may in addition increase the risk for another pemphigoid disease, namely mucous membrane pemphigoid (MMP), where exposure to these agents accounted for 24 cases of a total of 313 MMP patients in a recent French cohort study (70).

The clinical and immunological characteristics of DPP4i-related BP were described only in few studies. Izumi et al. (71) reported that seven patients with DPP4i-associated BP tended to present with the non-inflammatory phenotype, characterized by reduced erythema and scant lesional eosinophilic infiltration. Autoantibody reactivity against the midportion of BP-180, but not against the NC-16A immunodominant domain of BP180, was detected in these patients. Recently, Chijiwa et al. (72) reported that the frequency of lesional eosinophilia and mucosal involvement was significantly lower in nine patients with DPP4i-related BP than in 21 patients with non-DPP4i-related BP. Garcia-Diez et al. (73) had recently reported that 4 (50%) out of their 8 patients with DPP4-related BP developed non-inflammatory phenotype, whereas 6 (75%) were tested positive for anti-NC-16A BP180 autoantibodies in ELISA. In their longitudinal follow-up across 9 years, Kawaguchi et al. (74) found that 8 patients developed BP while being managed with DPP4i, of whom 6 (75%) had a non-inflammatory phenotype, and 5 of the 6 (83.3%) had been tested negative for anti-NC-16A BP180 autoantibodies in ELISA. The susceptibility to the development of DPP4i-associated BP was found to associate with HLA-DQB1^*^03:01 allele (75).

The pathomechanism underlying the association between DPP4i and BP has yet to be fully elucidated. It is known that DPP4 is a cell-surface plasminogen receptor that activates plasminogen, which leads to the formation of plasmin (76). The latter is a major serine protease that cleaves BP180 within the immunodominant NC-16A domain and can be detected in lesional skin as well as in blister fluid of patients with BP (77). The inhibition of plasmin by DPP4i may alter the appropriate cleavage of BP180, which may affect its antigenicity and function (71). Additionally, DPP4 inhibition may substantiate the activity of eotaxin and other pro-inflammatory cytokines, leading to cutaneous eosinophil activation and blister formation (78).

#### Psychotropic drugs

The intake of psychotropic medications, particularly phenothiazines with aliphatic side chains, was associated with an increased risk for the development of BP in two well-designed case-control studies (16, 79). The prescription of this group of medications has risen over the past few decades, according to studies across different countries (9, 80, 81). More alarming is the rise of polypharmacy of psychotropic medications within the last decade (80). Again, the mechanisms leading to psychotropic medication-induced BP remain unclear.

#### Checkpoint-inhibitors

The use of checkpoint inhibitors, such as therapeutic monoclonal antibodies targeting cytotoxic T-Lymphocyte Associated Protein 4 (CTLA-4), programmed cell death protein 1 (PD-1), and programmed death ligand-1 (PD-L1) has greatly improved the survival of many cancer patients, especially with metastatic melanoma (82). Due to the inhibition of immune checkpoints, chronic inflammatory, and autoimmune diseases are among the most common adverse events of checkpoint inhibitor treatment in cancer (83). This also includes the induction of BP under checkpoint inhibitor treatment. So far, at least 22 cases of BP have been reported under checkpoint inhibitor treatment (84, 85). While this most likely does not greatly add to the rising incidence of BP, the induction of autoimmunity toward BP180 may be a useful indicator to predict checkpoint inhibitor treatment outcomes (Poster LB1513 at the 2018 International Investigative Dermatology Meeting).

### Increasing awareness of the atypical variants of BP

Joly et al. (9) suggested that the improving awareness of the recently described clinical variants of BP, which were not recognized in the past, maybe another putative factor contributing to the increasing detection of BP. These BP variants that may appear in up to 20% of patients include the prurigo-like type (86), urticaria-like type (87), eczema-like type (88), dyshidrosiform type (89), erosive type (90), and erythema annulare centrifugatum-like type (30). In a recent systematic review of case reports and series that described BP patients lacking frank bullae, 132 patients with atypical non-bullous presentation had been identified. Urticarial plaques (52.3%) and papules/nodules (20.5%) were the most reported clinical features among these patients. Only 9.8% of patients developed bullae during the reported follow-up, thus leading to a substantial average delay of 22.6 months until a confirmatory diagnosis of BP (91).

Of interest, two previous studies reporting a growing incidence of BP revealed that a sizable portion of the patients presented with atypical clinical presentation. Joly et al. (9) found that 20.5% of their 502 BP patients had atypical clinical variants, of which the majority had prurigo-like and eczema-like variants. Kridin and Bergman (17) noted that 8.4% of their 287 patients had atypical clinical manifestation, with the urticaria-like and prurigo-like variants as the most common clinical picture seen among these patients.

This assumption is further supported by the recent survey conducted in the SSENIOR study (92). Herein the authors evaluated the prevalence of pruritus and pemphigoid in a high-risk population of nursing home residents. Almost half of the nursing home residents (48%) complained of pruritus. In addition. seven (6%) of 126 subjects had pemphigoid, of which four had chronic severe pruritus without blistering [non-bullous pemphigoid (91)], and three had known bullous pemphigoid. Hence, populations at risk, especially when pruritus is present, should be screened for the presence of BP using the appropriate measures. This further supports the assumption that the increased awareness has contributed to the increase in BP, but also highlights that it is likely that the incidence of BP will still increase, as approximately 4% of an at-risk population had undiagnosed BP.

### Better diagnostic methods

In 1953, Dr. Lever distinguished BP from pemphigus based on distinct histologic features, which had been not noted before (93). This distinction based on histomorphologic changes was a landmark discovery, allowing differentiating between pemphigus and pemphigoid. Another breakthrough discovery, which contributed to an improved diagnosis of BP, was the discovery of IgG deposits by direct immunofluorescent (IF) microscopy in skin specimen from BP patients (94). By evaluating direct IF microscopy at high magnification, patterns, such as n- or u-serration become visible. This allows distinguishing between different pemphigoid diseases. However, the BP-associated, n-serrated pattern, is present in BP and other pemphigoid diseases (95). Lastly, the presence of circulating anti-basement membrane autoantibodies in BP patient sera by indirect IF microscopy on human salt-split skin as a substrate was described (96). Indeed, in addition to H&E stained lesional biopsies, direct and indirect IF microscopy are sufficient to diagnose BP if the disease is suspected. However, these techniques are mostly provided by tertiary centers. To overcome this limitation, methods allowing screening for BP (and other autoimmune skin blistering diseases) have been developed in the past. First, autoantibodies against BP180 and BP230 can be detected by specific ELISA systems (97–99). In addition, paper-based ELISA systems have also been described, but are, not commercially available so far (100).

Second, if substrates for IF microscopy or ELISA are not available, biochips spotted with primate salt-split skin, recombinant BP180 and BP230 expressing cells can be purchased and used for indirect IF microscopy for the detection of circulating autoantibodies directed against BP180 or BP230 (101). This may be even automated, for high-throughput analysis (102, 103).

Taken together, direct IF microscopy of a perilesinal skin biopsy for the detection of tissue bound IgG/A and/or C3, and indirect IF microscopy on human salt-split skin for the detection of circulating anti-basement membrane autoantibodies are the minimal diagnostic criteria for BP. If these techniques are not available, specific ELISA systems and/or biochips may be used for BP diagnosis. Hence, a broad spectrum of diagnostic tests has become available, and thus certainly contributed to the increased incidence of BP.

## Conclusion

While BP is still a rare disease, its incidence rises exponentially in the elderly and is associated with remarkable burden. The growing incidence of BP warrants an expanded awareness of the disease, especially on its atypical non-bullous presentations. Alongside practicing dermatologists, other physicians caring for the elderly patients, including general practitioners and geriatricians, should be attentive of the increasing incidence of BP and to refer patients with a suggestive clinical picture to experienced centers. Caution and careful evaluation should be exercised with regard to the use of DPP4i, particularly in high-risk patients like those with disabling neurological conditions.

## Author contributions

All authors listed have made a substantial, direct and intellectual contribution to the work, and approved it for publication.

### Conflict of interest statement

The authors declare that the research was conducted in the absence of any commercial or financial relationships that could be construed as a potential conflict of interest.
